# Intramuscular hydatid cyst in the lower extremity: report of three cases

**DOI:** 10.1590/0037-8682-0255-2021

**Published:** 2021-08-20

**Authors:** Sadullah Şimşek, Salih Hattapoğlu

**Affiliations:** 1 Dicle University Medical School, Radiology Department, Diyarbakır, Turkey.

**Keywords:** Hydatid cyst, Hydatid disease, Echinococcosis, Cysts

## Abstract

Hydatid cysts are rarely detected in the muscle tissue, even in endemic countries. *Echinococcus granulosis* is the most common causative agent, whereas *Echinococcus alveolaris* is rare. Three patients, two females and one male, with primary echinococcosis of the skeletal muscles are described in this report. Mean patient age was 33.3 ± 14.6 years. The disease occurred as soft tissue masses in all three patients, and no hydatid foci were detected elsewhere in the patients. Skeletal muscle echinococcosis should be considered in the differential diagnosis of limb masses, especially in endemic countries.

## INTRODUCTION

Hydatid cysts are parasitic diseases transmitted to humans through dog feces; they are prevalent in countries wherein agriculture and livestock breeding are practiced. *Echinococcus granulosus* is the most common causative agent, while *Echinococcus alveolaris* is rare. In humans, hydatid cysts commonly affect the liver (55%-70%), followed by the lungs (18%-35%). The lungs and liver are simultaneously affected in 5%-13% of the patients^1^ ^,^ ^2^. Moreover, hydatid cysts may affect several organs. Hydatid cysts affecting the muscles account for approximately 1%-5% of all hydatid cysts^3^.

Hydatid cysts are rare in patients presenting with muscle swelling or muscle pain without swelling. This should be considered in the differential diagnosis for such patients. The diagnosis of a hydatid cyst should be ruled out, particularly in patients from endemic regions, before undergoing biopsy-aspiration to evaluate a possible abscess, hematoma, or tumor in the muscle. The present study aimed to present the findings of magnetic resonance imaging (MRI) to diagnose hydatid cysts and emphasize the need to consider hydatid cysts in the differential diagnosis of patients presenting with muscle swelling and pain.

## CASE REPORT

**FIGURE 1:**
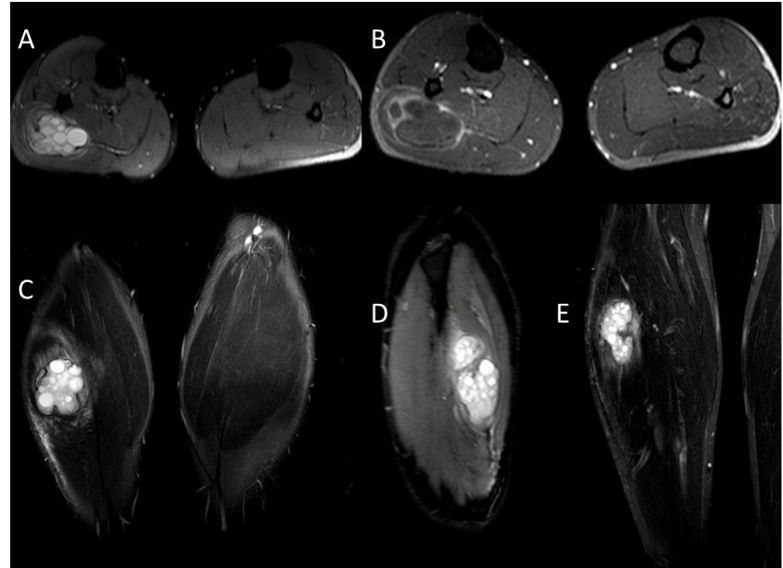
Contrast-enhanced images. Axial T2A **(A)**, post-contrast-enhanced axial T1A **(B)**, coronal T2A **(C)**, sagittal T2A **(D)**, post-contrast coronal-enhanced T1A **(E)** images showing a lesion compatible with a hydatid cyst in the soleus muscle.

Case 1: A 33-year-old male patient was admitted to the hospital with complaints of pain and swelling on the lateral side of the right calf. Complete blood count and biochemical tests revealed no abnormal findings. Her medical history was unremarkable, and there were no specific characteristics of the mass. MRI with contrast enhancement was performed for a pre-diagnosis of malignancy/lipoma, which revealed a 95 × 60-mm cystic lesion within the soleus muscle. The lesion appeared hypointense on T1-weighted images and hyperintense on T2-weighted images, suggesting the presence of multiple daughter cysts. Post-contrast-enhanced images demonstrated peripheral contrast uptake. Imaging results indicated a hydatid cyst diagnosis pathologically confirmed after surgical excision (Figure 1). No hydatid foci were detected elsewhere in this patient.

**FIGURE 2:**
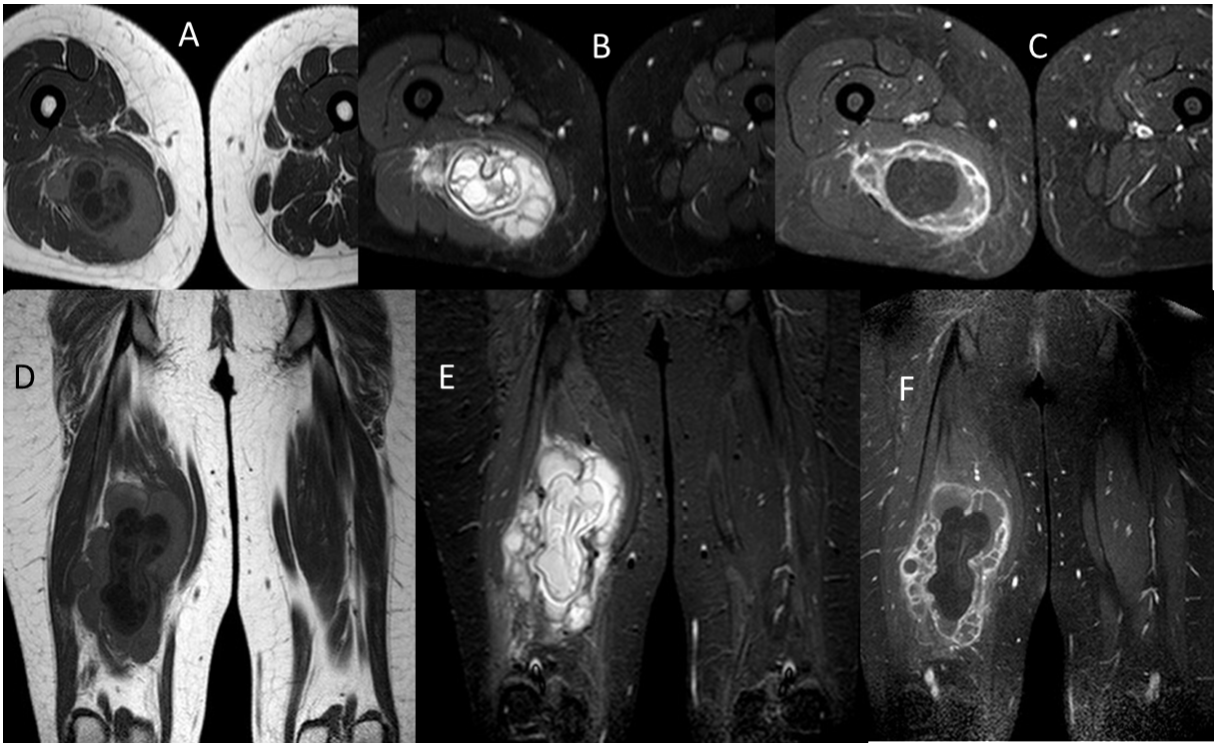
Post-contrast-enhanced images obtained by magnetic resonance imaging. Axial T1A **(A)**, axial T2A **(B)**, post-contrast-enhanced axial T1A **(C)**, coronal T1A **(D)**, coronal T2A **(E)**, post-contrast-enhanced coronal T1A **(F)** images showing a lesion compatible with a hydatid cyst in the adductor brevis muscle.

Case 2: A 26-year-old female patient was admitted to the hospital with swelling in the posterior region of the right thigh. Laboratory tests revealed no abnormal findings. Contrast-enhanced MRI revealed a 145 × 85-mm cystic lesion with membranous structures in the adductor brevis muscle. The lesion was hypointense on T1-weighted images and hyperintense on T2-weighted images. Peripheral contrast agent uptake was detected in post-contrast-enhanced MRI images, indicating a hydatid cyst diagnosis, which was confirmed with histopathological examination after surgical excision (Figure 2). No hydatid foci have been reported in previous studies.

**FIGURE 3:**
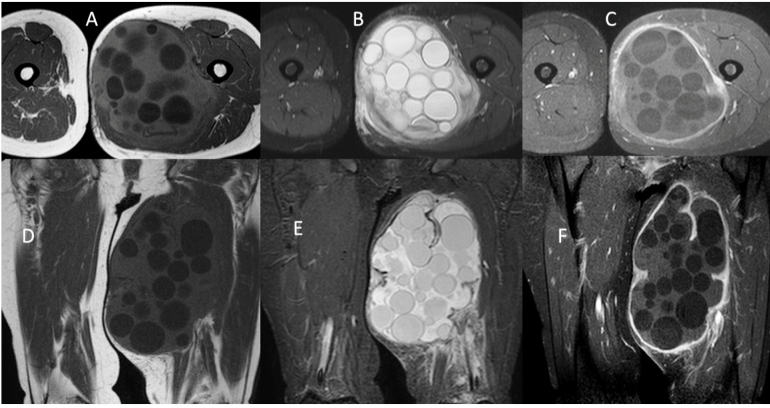
Post-contrast-enhanced images. Axial T1A (A), axial T2A (B), post-contrast-enhanced axial T1A (C), coronal T1A (D), coronal T2A (E), post-contrast-enhanced coronal T1A (F) images showing a lesion compatible with a hydatid cyst in the adductor brevis, sartorius, and semitendinosus muscles

Case 3: A 41-year-old female patient was admitted to the hospital with complaints of difficulty in walking. She complained of pain and swelling in the left thigh. Laboratory tests revealed no abnormal findings. Contrast-enhanced MRI was performed for the differential diagnosis of lipoma. MRI revealed a 200 × 130-mm cystic lesion with multiple daughter cysts in the adductor brevis, sartorius, and semitendinosus muscles. The cystic lesion was hypointense on T1-weighted images and hyperintense on T2-weighted images, with peripheral contrast agent uptake in post-contrast-enhanced images. Imaging characteristics indicated a hydatid cyst. The definitive diagnosis of a hydatid cyst was pathologically confirmed after surgery (Figure 3). There was no hydatid foci involvement except in the left thigh of the patient.

## DISCUSSION

Hydatid cysts are parasitic diseases caused by *E. granulosus* in 99% of cases and *E. multilocularis* in 1% of cases. Hydatid cysts are common in Southern Europe, Asia, Australia, Africa, and the Middle East. Animals such as dogs, wolves, and foxes are definitive hosts, and humans are intermediate hosts. The disease occurs after the consumption of food contaminated with live parasite eggs. Although hydatid cysts can be observed in almost all organs, they less commonly affect muscle structures. The thoracic wall muscles and pectoralis major, sartorius, psoas, quadriceps, and gluteus muscles are possibly affected by hydatid cysts. Psoas muscle involvement is reported as the most common intramuscular form of hydatid cysts^4^.

The pathogenesis of intramuscular hydatid cysts is not clearly understood. Two different theories have been proposed. The first theory suggests direct implantation owing to a dog bite, and the second theory suggests transportation through systemic circulation from the intestines to the skeletal muscle. Iatrogenic contamination of hydatid cysts can be considered if the musculoskeletal system and adipose tissues are involved after surgery^5^ ^,^ ^6^. 

The rare occurrence of hydatid cysts with primary muscle involvement is explained by the high lactic acid concentration in the skeletal muscles owing to its high activity, which prevents settling of the organism at this site^7^. Moreover, the barrier function of the liver and lungs is also important. It is considered that proximal muscles of the lower extremity are more commonly involved out of their large mass and rich blood supply.

The primary clinical sign of muscle hydatidosis is a localized and palpable mass. Additionally, patients can present with pain and restricted movement. Routine hemogram and biochemistry results are often normal. Although eosinophilia is anticipated in parasitic infections, it may not be observed in all patients.

The use of imaging methods such as ultrasonography (USG), computed tomography (CT), and MRI for the diagnosis of slow-growing cystic masses in the musculoskeletal system is important for accurate diagnosis. USG is the primary modality used to diagnose such patients. USG is a widely available, noninvasive, inexpensive and can be repeated. Cysts are classified according to the Gharbi criteria on USG (8). According to this classification, type 1 is defined as a pure cystic lesion, type 2 is a detached germinative membrane within the cyst, type 3 is a multi-cystic lesion separated into septate, type 4 is a degenerated cyst with a pseudo-solid view, and type 5 is a calcified pseudo-solid cyst. Daughter cysts, separated membranes, and double-line signs are the most characteristic features of hydatid cysts on USG^8^. While it is known that USG can be valuable in detecting hydatid cysts in skeletal muscles, it is emphasized that MRI findings are much more valuable. MRI is an important imaging method for the detection and characterization of soft tissue masses. As the MRI findings of muscle hydatidosis have not been well described as those of liver hydatidosis, it can be difficult to define these lesions. Additionally, CT is superior in examining wall calcifications, bones, and their relationship with neighboring structures. The view of the hydatid cyst on CT varies, and it rarely shows typical characteristics.

The presence of hydatid cysts should be considered when evaluating the lesions involving the muscles. Slow-growing tumors, hematomas, myositis, and abscesses should also be considered for differential diagnosis.

Diagnostic biopsy and aspiration should be avoided in cases of suspected hydatid cysts to prevent rupture and spread. Total cyst excision is the primary treatment method. Disease relapse is prevalent after surgical treatment. Postoperative mortality has increased from 0.95% to 3.5% owing to repeated surgical operations and accompanying disorders^9^.

In conclusion, a hydatid cyst must be considered in the differential diagnosis of a cystic mass with well-defined margins refractory to medical therapy in the extremities of individuals from endemic regions.
